# Cellular and Molecular Engineering of Glycan Sialylation in Heterologous Systems

**DOI:** 10.3390/molecules26195950

**Published:** 2021-09-30

**Authors:** Ryoma Hombu, Sriram Neelamegham, Sheldon Park

**Affiliations:** 1 Department of Chemical and Biological Engineering, University at Buffalo, Buffalo, NY 14260, USA; ryomahom@buffalo.edu (R.H.); neel@buffalo.edu (S.N.); 2 Center for Cell, Gene, Tissue Engineering, University at Buffalo, Buffalo, NY 14260, USA; 3 Department of Medicine, University at Buffalo, Buffalo, NY 14260, USA

**Keywords:** sialic acids, glycoproteins, pathway engineering, protein engineering, carbohydrate-active enzyme, cell free protein synthesis, therapeutic proteins, chemoenzymatic synthesis

## Abstract

Glycans have been shown to play a key role in many biological processes, such as signal transduction, immunogenicity, and disease progression. Among the various glycosylation modifications found on cell surfaces and in biomolecules, sialylation is especially important, because sialic acids are typically found at the terminus of glycans and have unique negatively charged moieties associated with cellular and molecular interactions. Sialic acids are also crucial for glycosylated biopharmaceutics, where they promote stability and activity. In this regard, heterogenous sialylation may produce variability in efficacy and limit therapeutic applications. Homogenous sialylation may be achieved through cellular and molecular engineering, both of which have gained traction in recent years. In this paper, we describe the engineering of intracellular glycosylation pathways through targeted disruption and the introduction of carbohydrate active enzyme genes. The focus of this review is on sialic acid-related genes and efforts to achieve homogenous, humanlike sialylation in model hosts. We also discuss the molecular engineering of sialyltransferases and their application in chemoenzymatic sialylation and sialic acid visualization on cell surfaces. The integration of these complementary engineering strategies will be useful for glycoscience to explore the biological significance of sialic acids on cell surfaces as well as the future development of advanced biopharmaceuticals.

## 1. Introduction

Whereas the synthesis of a polypeptide chain occurs predictably using a genetic blueprint, the post-translational addition of glycans on a protein is a non-templated process that follows loose guidelines with high variability. As such, significant heterogeneity exists among glycosylation products, both in the number of glycan groups that have been added, and the identity of each attached glycan. For example, O-glycosylation can occur through the addition of glucose, galactose, N-acetylglucosamine (GlcNAc), N-acetylgalactosamine (GalNAc), mannose (Man), or fucose on the hydroxyl side chain of serine, threonine, tyrosine, hydroxylysine, and hydroxyproline residues of cytoplasmic, nuclear, and extracellular proteins. This can result in a glycan chain containing from a single monosaccharide to a polysaccharide with many hundreds of such units, sometimes globally and at other times on specific domains or proteins. Another common form of glycosylation involves the attachment of bulky, branched oligosaccharides to the asparagine side chain within the consensus sequence N-X-S/T, where X is a non-proline residue. In mammalian cells, such N-linked glycosylation is also heterogeneous with a range of carbohydrate structures that can be capped by Man, GalNAc, Gal, or sialic acid (Sia). Thus, the glycan heterogeneity of N-linked sugars is more the norm than an exception.

A significant fraction of biopharmaceuticals today are recombinant proteins, and over 70% of these are known to be glycoproteins. Achieving humanlike glycosylation is important to preserve therapeutic activity, reduce immunogenicity, and ensure long serum half-life [1,2]. In this regard, sialylation is particularly relevant to the in vivo activity of a therapeutic protein, because sialic acids are often the most visible and accessible residues in a glycoform, owing to their terminal position [3]. Sialylation is also crucial for a long in vivo half-life, and non-sialylated proteins are recognized by hepatic asialoglycoprotein receptor (ASGPR or Ashwell–Morell receptor) and other receptors, resulting in their rapid removal from circulation [4,5]. Sialylation has been reported in many organisms and can be very different in different species. Of the many different sialic acids found in nature, N-acetylneuraminic acid (Neu5Ac) is exclusively found in humans. In mice, on the other hand, both Neu5Ac and N-glycolylneuramic acid (Neu5Gc) are part of sialylated epitopes. KDN (deaminated neuraminic acid) is another form of sialic acid found in nature [6]. Altogether, over 80 types of sialic acids have been reported in nature, and their distribution varies with organism and species [7]. Proteins bearing non-human sialic acids cannot be used in vivo because they are highly immunogenic. Since sialic acids are based on a nine-carbon backbone and carry a net negative charge, sialylation can significantly change the biophysical characteristics of the target molecule. For one, it can help stabilize intermolecular interactions by forming strong ionic bonds, whereas charge neutral monosaccharides rely on much weaker hydrogen bonds and van der Waals contacts. As sialic acids only occur as terminating units (unless they are part of a poly-sialic acid chain), sialylated glycoproteins on the membrane often play critical roles in the cell–cell communication that underpins cell trafficking, development, differentiation, immune activation, and host–pathogen interaction [8]. Mutations that affect sialylation have been linked to tumor metastasis, neurological disorders (such as Alzheimer’s and schizophrenia), and reduced immune response [9,10].

**Figure 1:**
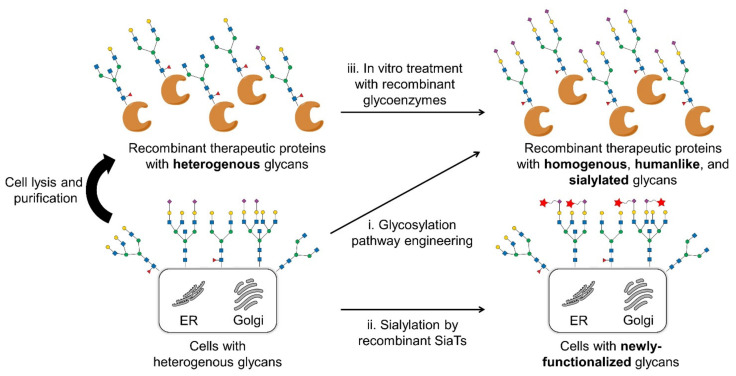
Strategies to achieve humanlike glycosylation through pathway engineering and in vitro sialylation. (i) Sialylation with Neu5Ac via pathway engineering or using recombinant SiaTs can reduce immunogenicity and increase the serum stability of recombinant proteins. Purified glycoenzymes, including SiaTs, may be used (ii) to functionalize cell-surface glycans, e.g., with a fluorescent dye and reaction handles, such as azido, alkyne, and biotin moieties, or (iii) to modify recombinant proteins to improve homogeneity and humanlike sialylation.

Recombinant expression of sialylated proteins in nonmammalian cells can be challenging if the organism lacks the enzymes for sialylation or naturally produces proteins with non-human sialylation patterns. Nonetheless, the high cost of maintaining mammalian expression systems continues to drive the search for simpler expression hosts and systems capable of synthesizing human sialoproteins [11]. In this review, we examine some attempts to achieve this expression goal in non-human cells (Figure 1). First, we describe the efforts to engineer a sialylation pathway in host cells by expressing heterologous enzymes or suppressing existing enzymes. Studies show that through targeted expression of essential enzymes the details of N-glycosylation and sialylation can be steered toward a single desired product. We review recent advances in model expression hosts, including Chinese hamster ovary (CHO) cells, bacteria, insects, and plants. Additionally, we discuss cell-free expression systems, in which key glycosylation pathways are reconstructed outside the cell in order to bypass some challenges inherent in cellular systems. As a complement to pathway engineering approaches, the second half of this review examines the studies in which the molecular properties of sialyltransferases (SiaTs) are dissected and engineered at a molecular level. These studies show that these enzymes can be engineered through rational design and directed evolution to modulate regioselectivity and perform sialylation reactions using unnatural Sia substrates. Moreover, these engineered enzymes are useful to advance glycoscience and study the role of sialic acids. In the future, engineered SiaT and glycoenzymes may also be used in vivo to achieve the predictable engineering of therapeutic glycoproteins; improving the efficiency and economics of current expression systems.

## 2. Strategies to Achieve Human Sialylation

### 2.1. Mammalian Cells

CHO cells are commonly used to express human therapeutic proteins; with baby hamster kidney cells BHK21 and human embryonic kidney cells HEK293 being used less often [12,13]. Therapeutic proteins, such as cytokines, enzymes, tissue factors, receptors, ligands, and antibodies, all undergo extensive N-linked glycosylation before secretion. A crucial modification for complex and hybrid N-glycans includes the addition of a sialic acid to terminal Gal residues. In this context, both the introduction of human Neu5Ac and an appropriate glycosidic linkage are important factors influencing in vivo characteristics.

#### 2.1.1. Enhancing the CMP-Sia Supply Chain Improves Sialylation

**Figure 2:**
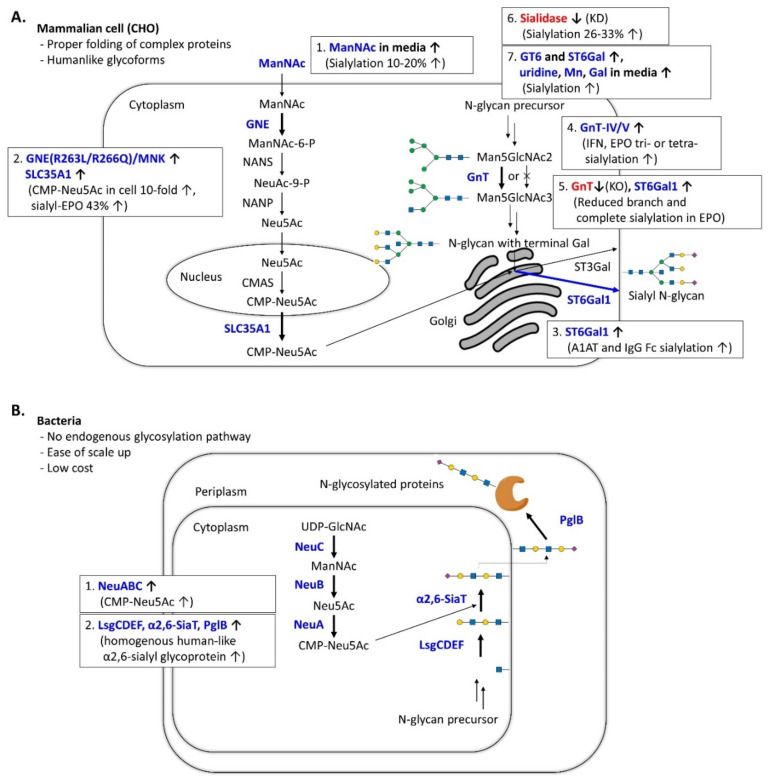

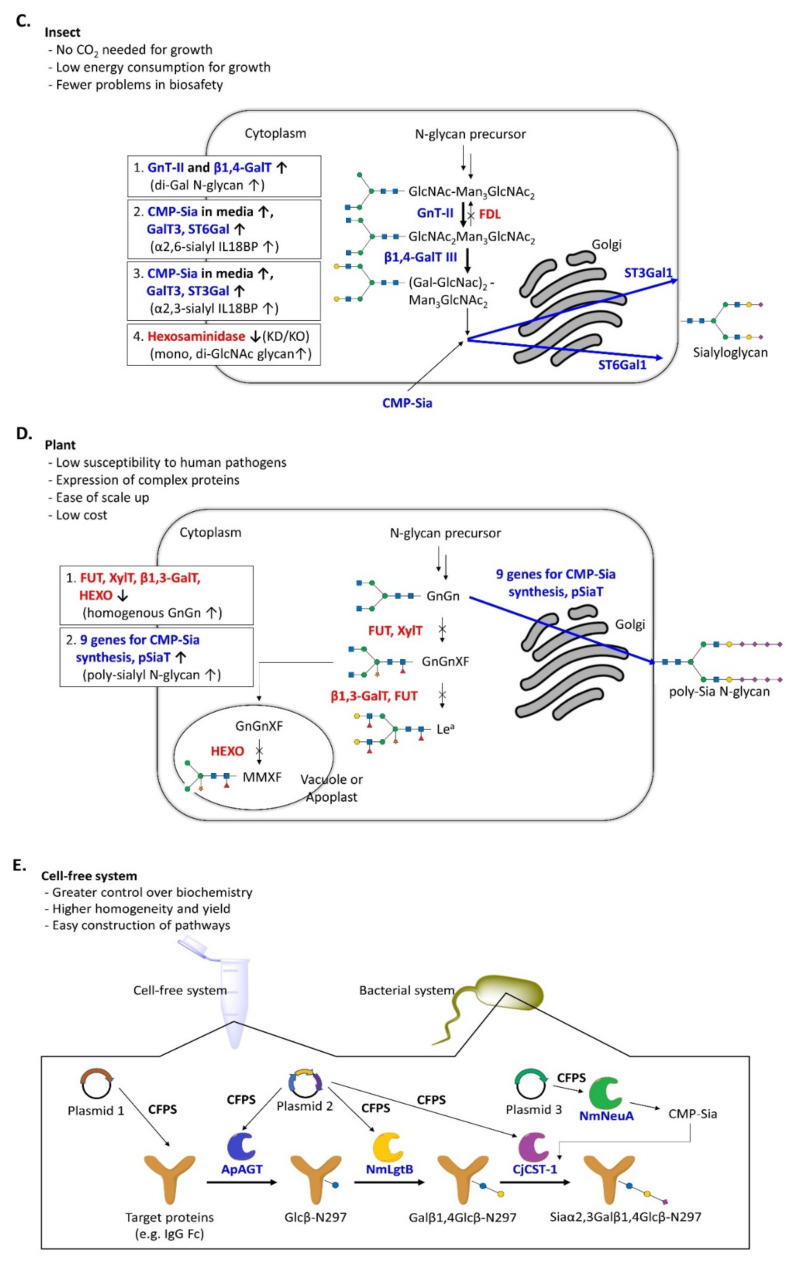
A brief description of the glycosylation pathway engineering strategies to achieve humanlike glycosylation in different expression systems: (**A**). mammalian cells, (**B**). bacteria, (**C**). insect, (**D**). plant, and (**E**). cell-free systems (cont’d on the next page). The advantages of using each host are listed. The enzymes or medium components enhanced via heterologous overexpression, knock-in, or media addition are shown in blue. The enzymes that were suppressed through knockout (KO) or knockdown (KD) are in red. Thick arrows indicate upregulation of the corresponding enzymes. KO/KD are indicated by arrows that are crossed out. Blue thick arrows indicate pathways which were newly introduced. The primary strategies utilized in individual papers are listed in square insets. See text for details.

In order to produce human like glycoforms, CHO cells have been engineered through gene knockout, heterologous enzyme expression, and media optimization (Figure 2A). Sialylation is a stochastic process and can occur with varying efficiency. One of the factors affecting this variability is the availability of CMP-Sia in the cells, as improving the efficiency of CMP-Sia delivery can improve the total sialylation level. Thus, supplementing media with 20 mM ManNAc was shown to increase sialylation by ~35-fold, indicating that sialylation can be externally controlled [14]. On the other hand, others have reported that nucleotide sugars are typically not rate limiting, since they are produced in cells at concentrations far greater than the K_M_ of glycosylating reactions [15]. Sialic acid is synthesized by a bifunctional enzyme that embodies two functionalities in one polypeptide chain: uridine diphosphate (UDP)–GlcNAc 2 epimerase (GNE), which is required for the synthesis of ManNAc from UDP-GlcNAc; and ManNAc kinase (MNK) activity, which produces ManNAc-6-PO_4_. Double mutations in GNE, R263L, and R266Q eliminate the known feedback inhibition of the enzyme, and when this mutant was co-expressed with CMP-Sia transporter (SLC35A1) in CHO cells, there was a 10-fold increase in the cellular content of CMP-Sia and a 43% increase in the sialic acid content of recombinant human erythropoietin (EPO) [16], which was accompanied by a 32% increase in the content of tetra-sialylated glycans and a 50% decrease in asialo or mono-sialylated glycans. This provides evidence that sialylation can be optimized through genetic engineering.

#### 2.1.2. Overexpression or Suppression of Glycoenzymes to Regulate the Extent and Stereochemistry of Sialylation

Sialyltransferases (SiaTs) transfer sialic acid from CMP-Sia to terminal galactose, GalNAc, or sialic acid residues on a variety of mammalian glycoconjugates [17]. There are four mammalian families of sialyltransferases: ST3Gal, ST6Gal, ST6GalNAc, and ST8Sia. Among these, the ST3Gal and ST6Gal enzymes are the most relevant to biotherapeutic production, as they transfer α2,3 and α2,6-linked sialic acids to terminal Gal on various glycoproteins. In this regard, while both α2,3 and α2,6 SiaT activities exist in most human cell types, only α2,3-linked sialic acids are found in CHO glycans, because hamster cells do not express functional ST6Gal enzymes. In order to humanize sialylation, ST6Gal1 was overexpressed in CHO while co-expressing tissue plasminogen activator (TPA) or interferon γ (IFNγ) [18,19]. Both expressed proteins contained sialoglycans with α2,3 and α2,6 linkage, which are more compatible for use in humans. Overexpression of ST6Gal1 was also successful in synthesizing therapeutically relevant recombinant proteins, including α1-antitrypsin (A1AT) and plasma protease C1 inhibitor with humanlike sialylation [20,21,22]. Other glycosyl enzymes were also expressed in CHO cells to induce the formation of multi-antennary glycoforms and promote sialylation. For example, overexpression of GnTIV/MGAT4 and GnTV/MGAT5 in CHO was shown to increase the tri and tetra-antennary glycan content of recombinant IFNγ and EPO to over 50% of the total sugar chains [23]. 

New glycosylation sites can be engineered by modifying the amino acid sequence to an N-linked glycosylation consensus sequence. Thus, EPO, leptin, and thrombopoietin receptor ligand were engineered with additional N-glycosylation sites to significantly increase their in vivo activity [24]. However, increasing N-glycans without achieving concomitant sialylation is undesirable because it exposes the underlying Gal residues and increases clearance via ASGPR. The large number of carbohydrate-associated enzymes expressed in cells makes it difficult to identify the most efficient strategy for achieving consistent sialylation. It is also possible that a biopharmaceutical requires homogeneous sialylation rather than a simple increase in the sialoglycan content to improve its bioactivity. To this end, knocking out MGATs, which control branching of N-glycans, can reduce the total number of sites that need to be sialylated, so that the remaining glycans can then be more fully sialylated. This strategy was implemented by knocking out the MGAT4A/4B/5 responsible for tri- and tetra-antennary branch formation. When ST6Gal1 was introduced through knock-in, complete sialylation of biantennary N-glycans was observed in CHO-produced EPO [22].

The final Sia content is determined by the interplay between SiaTs that add sialic acids, and sialidases that remove them. Since de-sialylation by sialidases can lead to incomplete modification of potential sialylation sites, one would expect that inhibiting the action of sialidases should increase net sialylation. Although sialidases are not secreted, they can be introduced into a medium through cells lysis [25]. When cell death was reduced through the removal of apoptosis inducers, there was an increase in the sialylated glycan content [26,27]. Likewise, targeting a CHO sialidase Neu1 and Neu3 with a short interfering and short hairpin RNA increased the sialic acid content by 26–33% [28]. However, even with a >90% suppression of the targeted sialidase activity, a redundancy in sialidase activities makes it difficult to prevent complete de-sialylation by targeting just one enzyme.

#### 2.1.3. Choice of Cell Lines for Transient Expression

The cell line plays an important role in determining the sialylation content. Two common cell lines used in the synthesis of biopharmaceuticals, CHO and HEK293, differ in their growth characteristics, protein titer, and glycosylation [29]. For instance, HEK293 cells are more efficiently transfected and start yielding proteins quickly. Transient transfection of CHO cells has a slow growth period, after which the titer begins to increase to a higher value. Both ST3Gal and ST6Gal enzymes are found in HEK293, whereas only ST3Gal enzymes are expressed in CHO, which leads to species-dependent sialylation products. The level of sialylation further varies with the transient or stable expression method used. In this regard, introducing galactosyltransferase-1 (GT1) or GT6 together with ST6Gal gene into the transient expression cell line ExpiCHO can facilitate branched N-glycan synthesis (e.g., a biantennary structure carrying 0–2 terminal Gal residues and a fucose: G0F, G1F, G2F) and sialylation. When supplemented with a medium containing uridine, manganese, and Gal, which act synergistically with expressed enzymes, ExpiCHO synthesized sialylated glycans at roughly half the rate of stable CHO cells [29]. Recapitulating the sialylation pattern of stable CHO in transient cell lines is relevant from a developmental perspective because transient expression is simpler and quicker to debug so that different protein variants can be tested quickly. This study shows that the characteristics of stable CHO, including sialoglycans, can be partially achieved during transient expression by modulating the glycoenzyme levels and the substrate concentration.

### 2.2. Bacteria

Microbial cells are an attractive protein expression platform because of its low production cost and ease of scalability compared to mammalian cells (Figure 2B). However, it is challenging to express authentic humanlike glycoforms in bacteria. Protein glycosylation exists in some bacteria, but the most commonly used bacterial species, *E. coli*, lacks a mammalian-like glycosylation pathway. On the other hand, this provides a clean slate for introducing a foreign glycosylation pathway to engineer a target protein with custom designed glycans. With the increasing knowledge of the glycogenes involved in glycan synthesis, including transporter, glycosidases, and glycosyltransferases, many enzymes have been exogenously expressed in *E. coli* to introduce a new glycosylation pathway and achieve customized glycosylation [30,31,32,33]. Some humanlike glycans, such as Lewis X (Le^X^) and Man_3_GlcNAc_2_, were thus engineered in *E. coli* by introducing corresponding glycoenzymes from other prokaryotes, such as the N-glycosylation pathway from *Campylobacter jejuni* [34], and eukaryotic organisms.

Engineered *E. coli* still does not fully replicate the glycosylation reactions in mammalian cells. For one, it lacks humanlike sialylation machinery and does not produce sialoglycans. Multiple additional steps are needed to engineer terminally sialylated N-glycoproteins acceptable for in vivo use [35,36]. Zhu et al. introduced glycosyltransferases LsgCDEF from *Haemophilus influenzae* and *C. jejuni* NeuBCA enzymes in *E. coli* to produce CMP-Neu5Ac [37]. These enzymes enable the construction of a sialylated glycan precursor, which is then transferred onto the protein target by another heterologously expressed enzyme, *C. jejuni* OGT PglB. N-glycoproteins with homogeneous sialylation and correct α2,6 linkage were thus obtained by expressing *Photobacterium leiognathid* α2,6-SiaT, demonstrating that it is feasible to engineer *E. coli* to synthesize therapeutically relevant human proteins.

### 2.3. Insects

Insect cells are commonly used to produce recombinant therapeutic proteins (Figure 2C). Some of the favorable characteristics of insect cells are the following: (i) they are able to express proteins of complex fold, (ii) do not require CO_2_ during growth, (iii) consume less energy due to their lower growth temperature, and (iv) pose less of a biosafety hazard [38]. Insect cells also produce more complex glycans than bacteria or yeast, potentially bringing their recombinant products closer to a therapeutic application [39]. The most widely used cell lines include *Drosophila melanogaster*, *Spodoptera frugiperda*, *Trichoplusia ni*, and *Bombyx mori* or silkworm. Due to the lack of a definitive analytical tool, there has been much debate regarding whether mammalian-like sialylation occurs in insects, but studies generally support that either sialylation does not occur in insects or is, at most, rare [40]. Mammalian glycoproteins, including cytokines, membrane receptors, and enzymes have been expressed in silkworms using a baculovirus expression system. However, the predominant N-glycans produced in insects are of paucimannose type, which are immunogenic and readily cleared from the circulation [41]. By supplying the sialic acid substrate from outside, the linkage and number of sialic acids were successfully controlled, significantly raising the possibility that silkworms may be capable of expressing high value biopharmaceuticals [42,43]. For example, when mammalian N-acetylglucosaminyltransferase GnT II and β1,4-galactosyltransferase III (B4GalT3) were co-expressed, di-galactosyl complex N-glycosylation was observed. Expressing B4GalT3 and ST6Gal1 while supplying CMP-sialic acid led to increased α2,6 sialylated glycans on co-expressed human IL18BP, as detected with the α2,6 specific lectin, *Sambucus nigra* agglutinin (SNA). Similarly, when ST3Gal3 was expressed instead of ST6Gal1, the expressed glycan was only detected by α2,3 specific *Maackia amurensis* lectin (MAL). Another way to improve humanlike glycosylation is to prevent the trimming of intermediate glycoforms to simple paucimannosidic structures by the action of β-N-acetylglucosaminidase (GlcNAcase) [44]. For example, suppression of the GlcNAcase function using RNA interference promoted the formation of complex glycans on heterologously expressed hEPO [45]. Likewise, CRISPR-Cas9 knockout of the Golgi GlcNAcase fused lobes (FDL), significantly increased the total content of hybrid type structures with one or two terminal GlcNAc moieties among cellular proteins [46]. Therefore, stereospecific sialylation can be introduced on mammalian proteins by expressing or suppressing appropriate enzymes. An advantage of the insect system compared to microbial cells is that fewer external genes are required to achieve sialylation. For example, the same process in yeast was much more daunting and required knocking out four endogenous genes and introducing 14 new genes [47].

### 2.4. Plants

Plants are used to produce glycosylated proteins because they are cheap to cultivate, able to produce complex proteins, pose a low risk of human pathogens, and can be easily scaled up [48]. The potential of a plant-based expression system is evident from the successful production of the ZMapp antibody used to treat Ebola [49] and flu vaccines [50] in engineered tobacco plants. Many plant proteins are known to be N-glycosylated, and, importantly, the initial steps of N-glycosylation are conserved between plants and mammals, including the presence of GlcNAc_2_Man_3_GlcNAc_2_ (GnGn), suggesting that plants may be used as general purpose expression hosts for therapeutic proteins [51]. However, genetic engineering of the glycosylation pathway is also needed, because plant glycans lack the complexity and diversity typically seen in mammalian cells. The most common glycan in plants is GnGn carrying β1,2-linked xylose and α1,3-linked fucose (GnGnXF) (Figure 2D). Similarly to insects, plants express paucimannosidic structures obtained by removal of the terminal GlcNAc from GnGnXF by acetylhexosaminidase HEXO, and Lewis A (Le^a^) structure obtained by the addition of glucose and fucose to the terminal GlcNAc [52]. Humanizing plant glycosylation pathways requires the simplification of these common plant glycans to GnGn through several coordinated adjustments. These include (i) the removal of plant specific xylose and fucose, (ii) suppression of HEXO activity, and (iii) removal of Le^a^. Through genetic engineering, several plant species of commercial interest were thus modified to achieve near homogeneous GnGn. Whereas genomic knockdown/knockout can trim unwanted residues to produce a barebone structure for reconstructing humanlike N-glycans, additional engineering is needed to achieve sialylation. Targeting relevant glycosyltransferases to the right compartment, such as the trans-Golgi network, is critical in this regard to yield the desired outcome [53,54]. Humanlike sialylation was achieved by reconstituting the sialic acid pathway through introduction of nine exogenous genes and α2,8 polysialyltransferase (pSiaT). Despite the complexity of the challenges, the engineered plants produced recombinant proteins containing poly-sialic acids; demonstrating that plants are amenable to a high degree of pathway engineering and modification.

### 2.5. Cell-Free Sialylation

There are challenges in reconstituting or modifying a glycosylation pathway in living cells, including the lack of control over the substrate availability, enzyme localization and expression level, interference with existing cellular pathways, and the potential toxicity of the reaction to the organism. In this regard, an orthogonal strategy has been developed to synthesize glycosylated and sialylated proteins outside the cell using cellular components that are assembled in a controlled manner [55,56,57]. The advantages associated with cell-free glycoprotein synthesis (CFGpS) include manipulation of the glycoforms, improved homogeneity and yield, facile design and rapid testing of alternative pathways, and improved economics (Figure 2E). Cell-free synthesis of proteins containing N-linked glycans is of particular relevance in the biomanufacturing of therapeutic proteins, because these glycans are well documented to be important for the pharmacokinetics and pharmacodynamics of biomolecules in vivo. CFGpS of N-linked glycans may proceed in several different ways, depending on how the glycan is added to the target Asn. The first step of N-glycosylation in mammalian cells involves an en bloc transfer of a branched glycan precursor from a lipid linked donor to a nascent peptide chain by oligosaccharyltransferases (OSTs). The enzyme can be supplemented by adding mammalian cellular extracts containing lipid-linked oligosaccharides (LLO) and OST. Alternatively, LLO and OST may be sourced from bacteria, which may be technically easier because bacterial OST are monomers and not a large protein complex, as in mammalian cells. Bacterial OST are also more promiscuous with regard to the donor identity, which allows different saccharide units to be introduced.

An important recent advance in CFGpS includes the discovery of a N-glycosyltransferase from *Actinobacillus pleuropneumoniae* (ApNGT) that transfers glucose from UDP-Glc to Asn, thus bypassing the need for LLO or OST [58]. The resulting GlcβAsn serves as an acceptor in the subsequent glycosylation reactions that append various monosaccharides, including Glc, Gal, or GalNAc. The glycan chain can be further elongated through repeated addition of GlcNAc, Fuc, or Sia [59]. Several enzymes capable of sialylation are known and attach Sia to terminal Gal with an α2,3 or α2,6 linkage. These same enzymes can also synthesize glycans carrying more than one Sia group. Sialic acids are easily accessible and often interact with protein receptors, which makes them an especially relevant residue in a glycan chain. In this regard, an engineered cell line, GlycoDelete, represents a minimalistic approach to glycoengineering, by replacing complex N-glycans with short linear glycans terminating with Sia. Surprisingly, the substitution can preserve in vivo functions of the original glycan, but using a much simpler structure [60]. Likewise, the short glycan chain synthesized during CFGpS has the potential to achieve homogeneous N-glycosylation on therapeutic proteins without compromising folding or efficacy. In addition to producing novel glycans in vitro, CFGpS can also guide the design of a glycosylation pathway in living cells. For example, CMP-Sia synthase (CSS), ApNGT, galactosyltransferase from *Neisseria meningitis* (NmLgtB), and *C. jejuni* SiaT (CjCST-1) expressed in *E. coli* can decorate the Fc fragment of an antibody on N297 with a trisaccharide, Siaα2,3Galβ1,4Glcβ, as expected.

## 3. Manipulating Sialyltransferases at a Molecular Level

Bacterial SiaTs (bSiaTs) belong to the GT38, GT42, GT52, or GT80 family of the CAZy classification (http://www.cazy.org/ (accessed on 1 September 2014), whereas all mammalian SiaTs (mSiaTs) belong to GT29 [61]. Based on their structure, GT42 enzymes have a single Rossman fold, a domain that binds nucleotides, and belong to the structural group glycosyltransferase (GT)-A; while the other enzymes belong to GT-B, which has two repeats of Rossman fold [62]. All mammalian SiaTs have a GT-A fold. Although GT-A enzymes typically require divalent cations (e.g., Mn^2+^ or Mg^2+^) for activity and contain a DxD motif to coordinate their binding, SiaT enzymes are peculiar in that they lack a DxD motif and have a metal-independent activity [63]. Functionally, bSiaTs are either α2,3 or α2,6 SiaTs or pSiaTs, although some are also promiscuous and capable of performing multiple types of reaction. Although there are significant sequence differences across the families or even within a family, all SiaTs share a similar catalytic mechanism. First, they all use CMP-Sia as donor for a sialylation reaction. Second, both bacterial and mammalian SiaTs are inverting glycosyltransferases, in which the stereochemistry at the C2 position of Sia is inverted during the reaction. Third, these enzymes use a histidine in the binding pocket as a catalytic base to deprotonate a hydroxyl group on the acceptor sugar and initiate a nucleophilic attack on CMP-Sia [64,65]. Inspired by the available structures, several interesting advances have been made to engineer SiaT enzymes for in vitro applications, including the chemoenzymatic synthesis of novel glycans.

### 3.1. Bacterial Sialyltransferases

#### 3.1.1. Controlling Hydrolysis and Sialidase Activity

Most SiaT engineering studies have been performed using GT80 enzymes because of the abundance of structural information (Table 1). The low yield of sialylated products is one of the challenges facing the use of bacterial SiaTs in biotechnology and is in part caused by the multifunctional nature of these enzymes. For example, in addition to transferring Sia from an activated donor to the target glycan, a SiaT may also catalyze hydrolysis of the donor substrate [66], remove Sia from sialylated products [67], and perform trans-sialidase reactions, in which a Sia group is moved from one sialylated carbohydrate to another acceptor substrate [68,69]. They may also catalyze a reversible or exchange reaction, and, in the presence of CMP and product, yield a SiaT acceptor and CMP-Neu5Ac [70,71]. These side reactions can interfere with the principal sialylation function of the enzyme and reduce the overall efficiency of the reaction. A detailed investigation of the enzyme kinetics of several SiaTs has been documented [71,72].

In some cases, distinct amino acids in the enzyme contribute to different activities, creating the possibility that one activity may be selectively suppressed without compromising other activities. For example, *Pasteurella multocida* SiaT (PmST1) catalyzes both α2,3 and α2,6 sialyltransferase reactions but also has a high intrinsic hydrolase activity that degrades the CMP-Sia donor substrate. Based on their structure, Sugiarto et al. hypothesized that mutations of M144 and A35 can shift the pKa of the catalytic residue D141 and alter the activity of PmST1 [73]. Consistent with this hypothesis, mutating M144 to D lowered the hydrolytic and sialidase activities by 20-fold and 5588-fold, respectively, without affecting the α2,3 SiaT activity. The crystal structure of the M144D mutant shows that the targeted loss of hydrolase activity is mediated by a conformational change induced by the mutation, which keeps the enzyme in an open conformation when bound to CMP-Sia [74]. The hydrolysis of donor substrates may also be avoided by excluding water molecules in the binding pocket. In this regard, the double mutations E271F/R313Y decreased the sialidase activity by 6333-fold, while maintaining the α2,3 SiaT activity by creating a hydrophobic environment in the activity pocket [73].

Reducing the intrinsic hydrolytic activity appears to be a general strategy for engineering a net gain in the sialyltransferase activity. This idea was further tested on α2,3 SiaT from *Photobacterium phosphoreum* JT-ISH-467 (Pph2,3ST), which was modeled using the complex tertiary structures of PmST1 bound to a pseudosubstrate CMP-3F-Neu5Ac and α-lactose. A sequence alignment with eight SiaTs from the GT80 family was also used to identify 13 amino acids for directed evolution studies. Together, the study identified A151D and L387A mutations, which individually reduced donor hydrolysis, while retaining α2,3 SiaT activity [75]. Changing the chemical environment around the catalytic base can predictably change the hydrolytic activity. To this end, the A235 to D mutation in α2,3 SiaT from *Photobacterium* sp. JT-ISH-224 (Psp2,6ST), which is structurally equivalent to the M144D of PmST1, reduced CMP-Neu5Ac hydrolysis by 2.6 fold [76].

Usually, the sialidase activity in bacterial SiaTs is regarded as an unintended side activity that needs to be suppressed. However, it may also be possible to reinforce this activity to engineer a novel sialidase with defined linkage specificity. The feasibility of this idea was demonstrated using α2,6 SiaT from *Photobacterium damselae* (Pd2,6ST), in which six residues important for substrate binding, sialyltransfer, and sialidase activity were first identified and then screened by saturation mutagenesis in a blue-white colony screening [77]. The sialidase reaction was specifically targeted during screening by supplementing the reaction with CMP [69]. The final triple mutant, S232L/T356S/W361F, had a 100-fold higher α2,6 sialidase activity and cleaved α2,6-Neu5Ac and Neu5Gc specifically, without hydrolyzing α2,3 or α2,8-Neu5Ac. Similarly, the sialyltransferase activity of α2,3 SiaT from *Pasteurella dagmatis* (PdST) was selectively reduced by 200-fold by H85D, while retaining the donor hydrolytic activity [78]. The mutation was postulated to work by weakening the enzyme–acceptor interaction, which was supported by a similar loss of α2,3 sialyltransferase activity in PmST1(H112A) [79]. These studies demonstrate that the catalytic activities in bacterial SiaTs can be manipulated using structural data to design novel enzymes.

#### 3.1.2. Changing Regioselectivity through Rational Mutations

**Figure 3:**
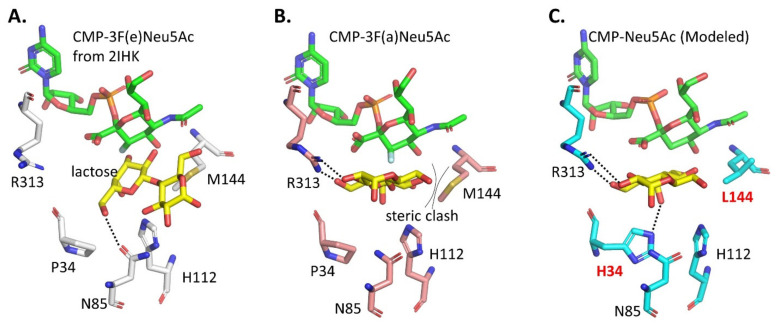
Rational engineering of PmST1 to change its regioselectivity. PmST1 exhibits both α2,3 and α2,6 SiaT activity. The structure of PmST1 bound to a donor analog and an acceptor suggests a design strategy to change the preferred regioselectivity of the reaction. (**A**). A ternary structure of PmST1 bound to the donor analog, CMP-3F(e)Neu5Ac, containing an equatorial fluorine at C3, and lactose was modeled from 2IHK (a binary structure of PmST1 bound to CMP-3F(e)Neu5Ac) and 2ILV (ternary structure of PmST1 with CMP and lactose). In this position, 3′ OH of lactose (yellow) is able to attack the anomeric carbon of the donor and form a α2,3-linked sialoglycan. (**B**). A ternary structure (2IHZ) of PmST bound to CMP-3F(a)Neu5Ac, containing an axial fluorine at C3, and lactose suggests a possible pre-catalysis complex that would lead to an α2,6 linkage. The bidentate hydrogen bonds from R313 stabilizes the bound acceptor, but M144 may create a minor steric clash with the galactopyranose ring of lactose. (**C**). The reaction may be biased toward α2,6 sialylation by favoring the orientation of lactose found in 2IHZ. Two mutations (red), P34H and M144L (or M144A), can simultaneously stabilize the acceptor through a new hydrogen bond with H34 (dotted line) and reduced steric clash. This structure was generated from 2IHZ by replacing 3F with hydrogen and introducing the mutations in PyMol before energy minimization.

Refining the regioselectivity of a SiaT can improve the activity of the enzyme by inhibiting competing reactions. To this end, the structures of PmST1 and Psp2,6ST with bound acceptor were analyzed to discover the determinants of regioselectivity [82]. In PmST1, P34 and M144 are responsible for creating an acceptor binding site that prefers the installment of Neu5Ac on the acceptor C3 for a α2,3 glycosidic bond. In Psp2,6ST, the same residue positions are occupied by H123 and A235, respectively, which leads to the acceptor binding in a conformation that favors the formation of an α2,6 bond. A sequence analysis among the GT80 family members revealed a correlation between regioselectivity and the conservation of these amino acid motifs. To confirm that these residues are indeed responsible for the observed regioselectivity, mutant PmST1 was created by substituting P34 with H, and M144 with A (Figure 3). The resulting double mutant lost the original regioselectivity and improved the α2,6-specific activity. As M144 is a known mutational hotspot among related proteins, other amino acids at the position were screened while retaining P34H. The optimized P34H/M144L double mutant was an effective α2,6 SiaT and showed a 50-fold lower sialidase activity compared to the wild type and a two-fold lower hydrolytic activity [81]. Similarly, Pd2,6ST(P7H/M117A) mutant exclusively formed an α2,6 bond, compared to the α2,3 activity of the wild type. When residues close to the acceptor and donor substrates of PmST1 were mutated, R313N/T265S were found to decrease the sialidase activity, while improving the α2,3 SiaT activity at the expense of the α2,6 activity [81]. Structural analysis suggested that R313N strengthens the interaction with the donor by reducing electrostatic repulsion. Together, these studies demonstrate that the regioselectivity of a SiaT is set by a few critical residues and may be altered through rational design.

Promiscuous installation of Sia can occur if the orientation of the acceptor is not well defined in the enzyme active site. Pd2,6ST can transfer one or two sialic acids to either monosaccharide in the T antigen (Galβ1,3GalNAc) because its active site is large enough to orient the acceptor in different ways. Controlling the degree of sialylation by regulating mono- or di-sialylation can produce a more homogeneous glycan structure, which is useful to study the biological role of sialylation. The movement of the acceptor can be constrained by reshaping the binding pocket, e.g., with a small to large amino acid substitution [90], to allow only certain bound conformations and, therefore, only one sialylation reaction to proceed. When A200Y/S232Y mutations were introduced in Pd2,6ST, the majority of reaction products were singly sialylated on the terminal Gal. In another study, Psp2,6ST was engineered to increase the di-sialylation in IgG Fc. To achieve this, the protein expression was first optimized using A366G, and then a mutational hotspot, A235, and nearby A203 were targeted for site-directed mutagenesis. The double mutant A235M/A366G identified from the screen was able to achieve a 73% di-sialylation of the anti-Her2 antibody trastuzumab [84].

#### 3.1.3. Controlling the Polysialylation Reaction

Polysialyltransferases of the GT80 family generate Sia oligomers containing an α2,8 or α2,9-linkage. To engineer a mutant with improved polysialylation activity, random pSiaT mutants from the *N. meningitidis* group B (NmPST) were expressed in *E. coli* EV36 lacking the endogenous pSiaT, NeuS [88]. Then, the poly-sialic acids (PSAs) displayed on the surface were quantified using GFP-EndoNF DM, which specifically binds PSAs [91,92]. After screening PSA displaying cells by FACS and a plate based assay for polysialylation of immobilized di-sialyl fetuin, a mutant containing four mutations (I360V, Y9S, E68V, and M340T) was identified that had a twofold higher poly-sialylation activity and was more stable than the wild type. However, because these mutations are located far from the active site it was not immediately obvious how they contribute to improved activity. Another NmPST mutant, K69Q, was identified using an *E. coli* strain, MB3109, lacking nanA (sialic acid lyase) and overexpressing CMP-Neu5Ac synthesis genes [93]. Interestingly, the mutant was able to synthesize PSA with a homogenous length [89]. Wild type NmPST is known to synthesize PSA using a processive mechanism, in which a PSA does not dissociate from the enzyme after each Sia addition and continues to serve as an acceptor for additional sialylation. The processive mechanism is the predominant mechanism as the glycan gets longer beyond six sialic acids [94]. The difference in affinity of PSA acceptor with distinct lengths results in heterogeneously elongated PSAs. On the contrary, NmPST(K69Q) uses a distributive synthesis mechanism, in which the PSA acceptor is released from the enzyme after the addition of each sialic acid. This is observed in relatively smaller oligo-sialic acids, containing fewer than six sialic acids. However, even longer PSAs can be extended equivalently by NmPST(K69Q), leading to the generation of homogenous PSAs, which in turn help create a therapeutic agent with more consistent molecular properties.

### 3.2. Mammalian Sialyltransferases

Mammalian SiaTs are grouped into four subfamilies, based on the linkage type they form: ST3Gal (I-VI), ST6Gal (I-II), ST6GalNAc (I-VI), and ST8Sia (I-VI). To date, crystal structures have been determined for porcine ST3Gal1 [65], rat ST6Gal1 [95], human ST6Gal1 [96], human ST6GalNAc2 [97], and human ST8Sia3 [98]. mSiaTs differ from bSiaTs in that they exhibit a higher fidelity of reaction and result in fewer side reactions, such as donor hydrolysis or trans-sialidase activity [71,99]. The higher acceptor specificity is useful for homogeneous synthesis of a sialylated product, although it can also be an obstacle when synthesizing novel glycans not found in nature. mSiaTs are often challenging to express in a microbial host, which has impeded their study. The first example of human SiaT in a bacterial host was reported in 2009, when active human ST6GalNAc1 was obtained from an engineered strain of *E. coli* [100]. The yield was improved by fusing it with maltose binding protein (MBP) and expressing the fusion in an oxidative *E. coli* strain, such as Origami, to allow the formation of disulfide bonds. Similar studies were repeated later, to express human ST3Gal1 and ST6Gal1 in *E. coli* [101]. Although MBP fusion increases solubility, the N-terminal affinity tag was not always accessible, suggesting expressed mSiaT may aggregate. In this regard, introducing surface mutations to increase the surface charge and create electrostatic repulsion was shown to increase the yield. mSiaT was also expressed in methanotrophic yeast. For example, a truncation mutant of ST6Gal1, Δ108ST6Gal1, was purified from *Pichia pastoris* KM71H and was able to transfer Sia to asialofetuin and IgG1 Fc [99]. The yield was optimized by changing the medium composition and mutating potential sites of proteolysis [102].

### 3.3. Measuring Sialyltransferase Activity

SiaT activity is typically measured using HPLC [103,104] and mass spectrometry [105] because they can inform about the regioselectivity of the reaction, provide a detailed description of the product glycan structure, and detect side reactions. However, the low-throughput nature of these assays is a significant obstacle for engineering a novel variant, since finding a rare mutant requires screening a large number of mutants and thus demands an inherently high throughput detection system. For example, the human glycome may be systematically knocked out to study each enzyme and to create a genetic background [106,107]. One high-throughput detection system uses fluorescently tagged CMP activated Sia [108]. Alternatively, biotinylated Sia may be used with streptavidin to investigate the substrate specificity of mSiaT [109]. Chemically modified Sia containing an alkyne or azide tag may be advantageous in some situations due to their smaller size. Noel et al. explored the substrate preference and the role of divalent cation in ST3Gal1 and ST6Gal1 using CMP-Sia alkynylated at C5 acetamide [110], which was used to introduce a Sia-specific biotin group in a click reaction with azido-biotin.

For the detection of PSA, a mutant of neuraminidase without glycosidase activity, EndoNF DM, fused to GFP may be used. The detection of PSA on the *E. coli* surface [88] and microplate [92] was thus reported. PSA on cells can also be detected by hydrolyzing Sia residues to generate soluble monomers, which are then quantified by attaching 1,2-diamino-4,5-methyleneoxybenzene (DMB) to the α ketocarboxylic acid at the reducing end [111]. However, this method necessarily destroys the target glycan and the detection of the reaction product requires HPLC analysis, which is difficult to scale. Another method that has been reported for regiospecific sialylation uses *p*-nitrophenol (*p*NP) conjugated glycan. For example, to change the regiospecificity of PmST1 from α2,3 to α2,6, PmST1 mutants were allowed to sialylate Galβ*p*NP before the sialylated glycan was treated with α2,3 sialidase and β galactosidase. Only α2,3 linked Sia is removed and processed by β galactosidase to give free *p*NP, whereas α2,6-linked Sia protects the glycan from further degradation. The resulting free *p*NP was converted to *p*-nitrophenolate and quantified [81].

Measuring SiaT activity inside a cell can be useful for studying sialylation during cellular processes, e.g., cell division. To detect sialoglycan, Bao et al. developed a fluorescence resonance energy transfer (FRET) sensor using 3-aminophenylboronic acid (APBA) that forms a stable complex with Sia [112]. APBA and asialofetuin, a well-known substrate for many SiaT, were delivered to cells in a liposome. Each molecule was fused to a FRET acceptor and donor, respectively, so that there is increased energy transfer when AF is sialylated. For SiaT displayed on the cell surface, e.g., on the outer membrane of *E. coli*, the typical biochemical assay based on product quantification may be used to measure activity [113]. A similar assay may be used for other cell display platforms, such as yeast display, during engineering and optimization studies.

### 3.4. Applications of Engineered Sialyltransferases

**Scheme 1:**
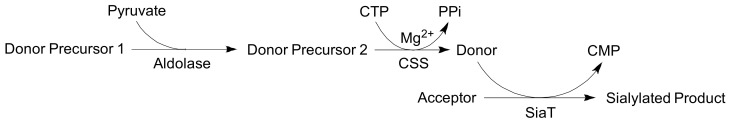
One-pot multienzyme system for synthesizing sialylated products from precursors and glycoenzymes.

Engineered or wild-type SiaTs can be good catalysts to attach sialic acid or its derivatives to an acceptor carbohydrate. However, it is important to calibrate the regioselectivity, catalytic efficiency, and substrate specificity of an enzyme before its use in a specific biotechnology application. For example, mSiaTs have higher substrate specificity and regioselectivity and are ideal for introducing a precise modification, e.g., α2,3-, α2,6-, or α2,8-linkage, when other competing reactions exist. On the other hand, bSiaTs are promiscuous and may be suited for generating glycan structures not found in nature (Scheme 1, Table 2).

### 3.5. In Vitro Chemoenzymatic Carbohydrate Synthesis

Free carbohydrate molecules with unnatural moieties or an unusual branching pattern can be physiologically active yet metabolically stable, making them potentially useful for in vivo applications [114,115]. They may also be included in a glycan array to study glycoenzymes and glycan binding proteins [116]. Although there have been advances in the chemical synthesis of glycans, the technology remains time-consuming, inefficient, and often complex. Instead, glycosyltransferases and glycosidases may be combined to synthesize glycans with a desired linkage with high efficiency [117]. For example, PmST1 was used to synthesize glycosylated vancomycin and pseudo-vancomycin carrying unnatural glycans [118]. PmST1(M144D), described previously, has reduced sialidase and donor hydrolysis activity and was recently used in chemoenzymatic synthesis of sialylated products from an array of donor and acceptor substrates [67]. The enzyme tolerates variations in the acceptor and is able to sialylate LacNAc, Le^X^ (Galβ1,4(Fucα1,3) GlcNAc), Le^a^ (Galβ1,3(Fucα1,4)GlcNAc), and O-sulfated Le^X^ (Galβ1,4(Fucα1,3) GlcNAc6S) [119,120], which makes it good for generating diversity. Likewise, donor substrates carrying C5 acetamide, C5 trifluoroacetamide, C9 hydroxyl, and C9 acetamide were also transferred by the enzyme, although a variation in efficiency was observed. Other bSiaTs mutants, such as wild type Pph2,3ST, Psp2,6ST(A235M/A366G), and Pd2,6ST(A200Y/S232Y), recognize C5 phenylacetamide, C5 levulinoyl group, and longer or branched acceptors [75,84,87]. Several studies have also demonstrated that modifications at C7 and C8 do not impede catalysis by many bSiaT enzymes. For example, C7 mono-fluorinated or di-fluorinated sialic acid is recognized by PmST1, Psp2,6ST, as well as Cst-I and Cst-II from *C. jejuni*, which were used to sialylate glycoprotein A1AT to assess their stabilizing effects [121]. Neu5Gc methylated in the C8 hydroxyl group was attached to terminal GalNAc through α2,6 linkage by PmST1 and Psp2,6ST, whereas replacement of the C5 acetamide on terminal GalNAc with a smaller azide enabled α2,3 sialylation [122]. Legionaminic acid, a sialic acid with C7 acetamide and no C9 hydroxyl group, was efficiently transferred by Psp2,6ST(A235M) onto fluorescently labelled LacNAc [123].

mSiaTs are used in chemoenzymatic carbohydrate synthesis, where high substrate and regiospecificity is required to ensure the synthesis of a single unique product (Table 3). For example, a combination of human ST3Gal1 and ST6GalNAc5 was used in chemoenzymatic synthesis of a di-sialyl glycolipid, DSGb5 [124]. These enzymes specifically catalyze α2,3 sialylation to terminal Gal and α2,6 sialylation to GalNAc, respectively. Although the chemical synthesis of di-sialylated glycolipid was also reported, chemical synthesis requires several deprotection steps after each sialylation, which is time-consuming and lowers the yield [125]. It was shown that sialylated multi-antennary *N*-glycans can be synthesized by human ST6Gal1 and ST3Gal4, each of which recognizes LacNAc as acceptor [126]. Branch selective sialylation by ST6Gal1 is a notable feature in terms of the substrate specificity that may be used to create custom designed mono- and di-sialylated proteins and accomplish asymmetric *N*-glycan synthesis [127,128]. A sialic acid containing alkynyl group in C5 acetamide was transferred by ST3Gal1 and ST6Gal1, suggesting that mSiaTs may be useful for introducing modified Sia in some cases [129,130]. ST6Gal1 was also good at attaching other modified Sia on a LacNAc acceptor, including Neu5Ac-9Az and Neu5Ac-9SNSydCl, containing an azide and sydnone moiety instead of the C9 hydroxyl group, respectively [131]. Sia containing C7 fluoride, Neu5Ac-7F was transferred to the glycoprotein asialo-A1AT by ST6Gal1 [121]. Further expanding the donor variety, legionaminic acid was used as a donor substrate by porcine ST3Gal1 and human ST6Gal1 for several acceptors [132]. When analyzing the yield from chemoenzymatic glycan synthesis, it is important to note that many of these studies were done using a one-pot multienzyme (OPME) system, in which SiaT and CMP-Sia synthase were added together, and the apparent efficiency of the reactions reflects the combined efficiency of SiaT and nucleotide sugar synthase [131].

### 3.6. Modification of Glycans on Cell Surface

Characterizing the glycan expression pattern, localization, and abundance is essential for research and therapeutic applications. Mass spectrometry is a powerful tool for detecting and analyzing cell surface glycans [133,134], but the technique is destructive. Alternatively, the glycans may be tagged with a fluorescent molecule by bacterial and mammalian SiaT, so that they can be visualized in situ (Table 4). For example, PmST1(M144D) and Pd2,6ST, as well as two fucosyltransferases, were used append Cy3 or biotin-labeled Sia to LacNAc on the cell surface [108]. The dye may also be introduced by installing Neu5Ac with C5 azide-bearing acetamide and performing click chemistry with an alkynylated biotin or dye. Cst-II was used to attach a BODIPY-tagged sialic acid [135]. Sialyltransferases may also be used to attach a PSA polymer, as demonstrated using NmPST in vitro and in vivo [136]. The installment of PSA on a neuronal cell adhesion molecule (NCAM) affected cell–cell adhesion, neurite outgrowth, and cell migration [137], which is consistent with the observation that NCAM is a major target of polysialylation and undergoes a change in PSA decoration during neural development [138].

mSiaTs have also been used to modify cellular glycans. For example, ST3Gal1, ST6Gal1, and ST6GalNAc4 were used to decorate HeLa cells with fluorescent Neu5Ac [140]. All three SiaTs can utilize CMP-Neu5Ac modified by Alexa Fluor 555 in the C9 position. Using fluorescent labeling, the precise localization of *N*-glycans and *O*-glycans on HeLa cells can be analyzed. Neu5Ac derivatives modified by BODIPY in the C5 and C9 positions were recognized by human ST3Gal1 and ST6Gal1 for labeling Jurkat cells [135]. ST6Gal1 is tolerant of donor modification and efficiently transfers Sia carrying large substituents at C5 acetamide, such as heparan sulfate or biotin, which is useful for introducing direct cell surface modification following sialidase treatment and can be used to modulate cell signaling or enable detection by avidin reagents [139]. Toward demonstrating the utility of recombinant SiaTs in clinical applications, treatment with galactosyltransferase B4GalT1 and ST6Gal1 improved the clinical score in autoimmune disease model mice by increasing the sialylation of IgG Fc, specifically at the sites of inflammation, such as the joints and kidneys [141].

## 4. Conclusions

Extracellular proteins are heavily glycosylated for many reasons, including the beneficial effects of glycans on protein folding, as well as their direct involvement in biological activities. In this regard, sialic acids play an oversized role among glycan subunits in determining the stability and efficacy of a therapeutic protein. This is because of their placement at the end of a glycan, where they are easily seen by other molecules and can critically influence intermolecular interactions. Therefore, it is imperative to achieve proper sialylation when preparing recombinant proteins for therapeutic application. Many expression hosts in use today, both simple and complex, lack the proper cellular machinery to correctly implement the necessary glycosylation and sialylation. For example, the required enzymatic pathway may be missing in some organisms, while others may attach glycans that are demonstrably different and, thus, highly immunogenic. An aspirational engineering goal is thus to develop an expression system that can produce recombinant proteins with correct humanlike sialoglycans and to do so while keeping the cost of production and the potential of pathogen transmission low.

In this review, we have examined several such systems in development, highlighting recent efforts to introduce novel glycosylation pathways in select hosts to reproduce humanlike sialylation. An important lesson from these studies is the remarkable plasticity of these systems. It is not unusual to knockout multiple endogenous proteins while introducing foreign enzymes, yet not only does the host survive such large-scale genomic engineering unscathed, but the effects of genetic manipulation can also be predicted. Thus, through deductive reasoning one can build more and more complex systems by optimizing each step and building on earlier accomplishments. The examples herein should thus lay the foundation for future elaboration, in which additional genes are targeted to achieve even more authentic sialylation in recombinant products. In the second half of the review, we discussed the molecular engineering of sialyltransferases. These enzymes have highly specialized functions and some common features have been identified from extensive structural and biochemical studies. The engineering examples included in this review highlight the advances in our understanding of the structure–function relationship in SiaTs, as illustrated by the successful optimization of enzyme properties through structure-based mutations. The engineered SiaT enzymes should spawn immediate opportunities in the lab and in the marketplace, through the chemoenzymatic synthesis of novel chemicals. They can also feed into pathway engineering to further improve the recombinant products that are being developed in nonmammalian hosts. The promise of bringing the two parallel disciplines into one, sharp focus is alluring, as it will help create high quality biotherapeutics with fine-tuned molecular properties and unprecedented efficiency.

## Figures and Tables

**Table 1:** List of bSiaTs discussed in this review, including their 3D structures and examples of engineering.

SiaT	Organism	CAZy Group	Regio-Selectivity	3D Structure (PDB)	Ref. for Structure	Examples of Engineering (Mutation and Improvement)	Ref. for Engineering
PmST1	*Pasteurella* *multocida*	GT80	α2,3 and α2,6			M144D, lower donor hydrolysis and sialidase activity (20- and 5588-fold, respectively)	[67]
						E271F/R313Y, lower sialidase activity (6333-fold)	[67]
				WT (2EX0, 2EX1, 2IHK, 2IHJ, 2IHZ, 2ILV, 2IY8, 2IY7, 2C84, 2C83)	[64,67,80]	P34H/M144L, converted regioselectivity to α2,6 and lower donor hydrolysis and sialidase activity (2- and 53-fold, respectively)	[81]
				M144D (3S44)			
	
						R313X (X = N, T, Y, H, D), converted regioselectivity to α2,3	[79]
						R313X/T265S (X = N, H), converted regioselectivity to α2,3	
						and higher α2,3-SiaT activity	
PdST	*Pasteurella* *dagmatis*	GT80	α2,3	WT (4V2U)	[82]	P7H/M117A, converted regioselectivity to α2,6	[82]
				P7H (4V38, 4V3B)			
				P7H/M117A (4V39, 4V3C)			
PspST	*Photobacterium sp.* JT-ISH-224	GT80	α2,6	WT (2Z4T)	[83]	A235D, lower donor hydrolysis (2.6-fold)	[76]
						A235M/A366G, improved antibody di-sialylation	[84]
PphST	*Photobacterium phosphoreum* JT-ISH-467	GT80	α2,3	WT (2ZWI)	[85]	A151D, lower hydrolysis and sialidase activity	[75]
						(4- and 68-fold, respectively)	
						L387A, lower hydrolysis and sialidase activity	
						(10- and 68-fold, respectively)	
Pd2,6ST	*Photobacterium damselae*	GT80	α2,6	WT (4R83, 4R84, 4R9V)	[86]	S232L/T356S/W361F, higher α2,6-sialidase activity (100-fold)	[81]
						A200Y/S232Y, converted regioselectivity to terminal sialylation	[87]
NmPST	*Neisseria meningitidis* group B	GT38	α2,8	No structure available		I360V/Y9S/E68V/M340T, higher stability	[88]
						and pSiaT activity (2-fold)	
						K69Q, pSiaT activity for PSAs with homogenous length	[89]

**Table 2:** Examples of in vitro chemoenzymatic sialylation achieved with bSiaTs or their mutants. Acceptor substrates, donor substrates, or their precursors used in OPME systems, sialylated products, derivatized moieties (R1-R5), yields, and references are also listed. The abbreviations are as follows: CTP, cytidine triphosphate; CSS, CMP-sialic acid synthetase; CMP, cytidine monophosphate; Pro, n-propyl; NHAc, N-acetyl; NHGc, N-glycolyl; NHAcN3, 2-azido-N-acetyl; NHTFA, 2,2,2-trifluoro-N-acetyl; FL, (Z)-10-(5-(4-((but-2-en-1-yloxy)methyl)-1H-1,2,3-triazol-1-yl)pentyl)acridin-9(10H)-one; NHAcPh, 2-phenyl-N-acetyl; NHLev, N-levulinoyl; oNP, o-nitrophenol; MU, 4-methylumbelliferyl alcohol; Hex, n-hexyl; FCHASE, 6-(5-fluorescein-carboxamido)-hexanoic acid amide; Leg, legionaminic acid. (Next page.).

SiaT	Acceptor	Donor Precursor or Donor	Sialylated Product	R^1^	R^2^	R^3^	R^4^	R^5^	Yield	Ref.
PmST1(M144D)	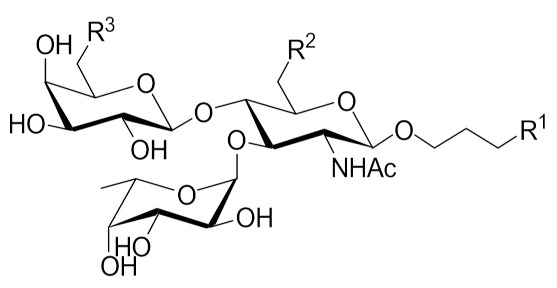 Le_x_βProR^1^	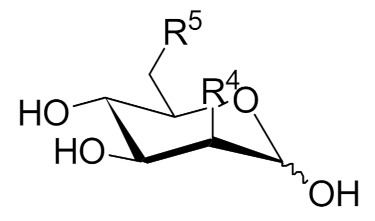 Donor Precursor 1	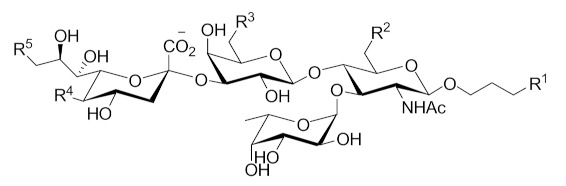 sLe_x_βProR^1^	N_3_	OH	OH	NHAc	OH	93	[67]
							NHGc		87	
							OH		85	
							NHAcN_3_		89	
							N_3_		84	
							NHAc	N_3_	91	
								OAc	62	
							NHGc		64	
		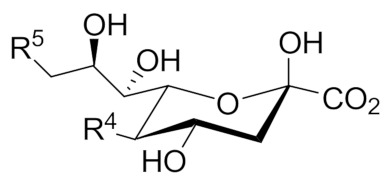 Donor Precursor 2		N_3_	OSO_3_H	OH	NHAc	OH	85	[120]
				NH_2_	OH	OSO_3_H			47	
					OSO_3_H				82	
				N_3_	OSO_3_H	OH	NHGc		60	
				NH_2_	OH	OSO_3_H			64	
					OSO_3_H				38	
	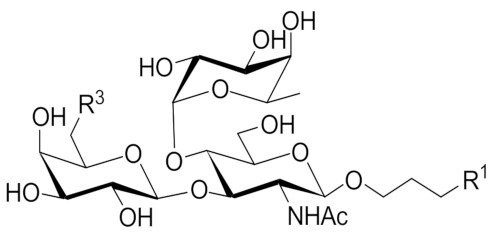 Le_a_βProN_3_	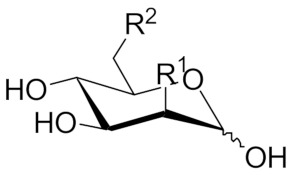 Donor Precursor 1	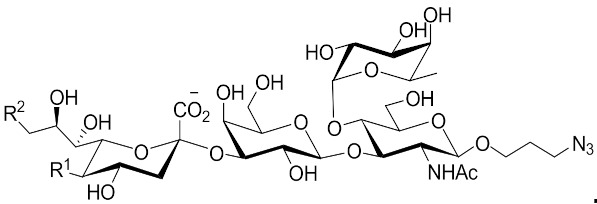 sLe_a_βProN_3_	NHAc	OH				85	[119]
				NHGc					82	
				OH					86	
				NHTFA					80	
				NHAc	NHAc				76	
		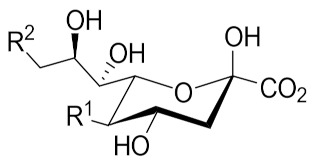 Donor Precursor 2		NHAc	OAc				62	
				NHGc					51	

PphST	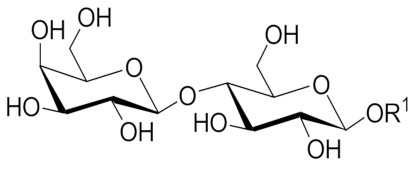 Lac	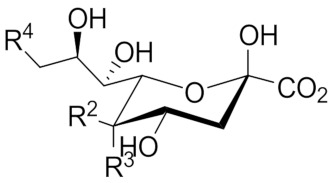 Donor Precursor 2	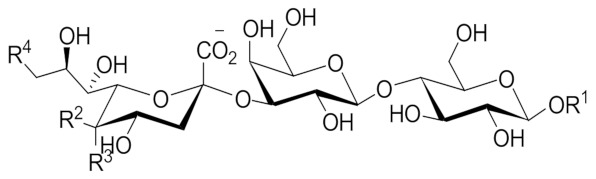 Neu5Acα2,3Lac	FL	NHAc	H	OH		ND	[75]
					NHGc					
					OH					
					H	OH				
					NHAc	H	N_3_			
					NHAcPh		OH			
PspST (A235M/A366G)	Herceptin (G2F glycoform)	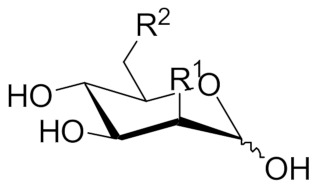 Donor Precursor 1	Herceptin (A2F glycoform)	NHAc	OH				ND	[83]
				NHAcN_3_						
				NHLev						
				NHAc	N_3_					

CjST1	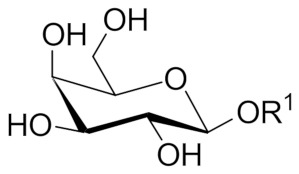 GalβR^1^	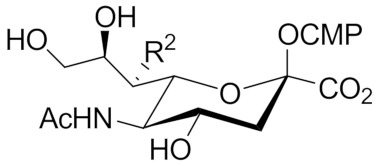 Donor	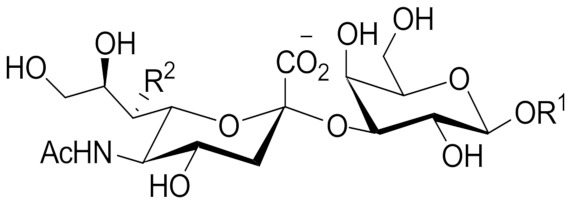 Neu5Acα2,3GalβR^1^	oNP	OH				75	[121]
					F				64	
				MU	OH				65	
					F				51	

PmST1	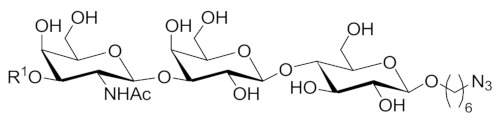 GalNAcβ1,3Galβ1,4GlcβHexN_3_	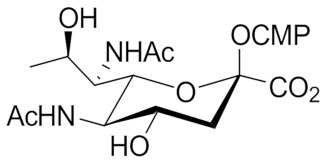 Donor Precursor 2 (Neu5Gc8Me)	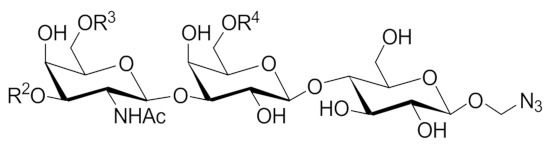 sialyl GalNAcβ1,3Galβ1,4GlcβHexN_3_	H	H	Neu5GcMe	H		70	[122]
PspST									88	
PspST				Neu5Gc8Me	Neu5Gc8Me	Neu5GcMe	H		77	
Pd2,6ST						Neu5Gce	H		30	
						H	Neu5Gc8Me		20	
PspST(A235M)	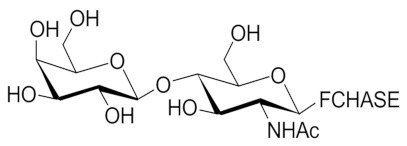 LacNAc-FCHASE	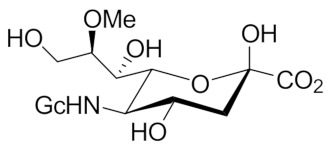 Donor (Leg5Ac7Ac)	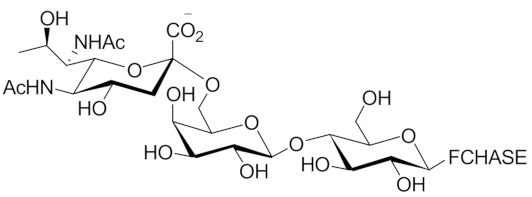 Leg5Ac7Acα2,6LacNAc-FCHASE	Regioselectivity for product was not explored					~30	[123]

**Table 3:** Examples of in vitro chemoenzymatic sialylation achieved by mSiaTs. Acceptor substrates, donor substrates, sialylated products, derivatized moieties (R1), yields, and references are also listed. The abbreviations are as follows: MS, mono-sialyl; DS, di-sialyl; Pen, pentyl; Cbz, benzyloxycarbonyl; PhSydCl, 3-(4-carbamoylphenyl)-4-chlorosydnone; Cer, ceramide; A1AT, α1-antitrypsin. (Following page.).

SiaT	Acceptor	Donor Precursor or Donor	Sialylated Product	R^1^	Yield	Ref.
ST3Gal1	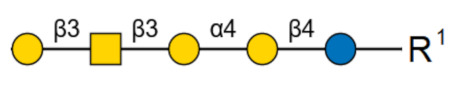 Gb5	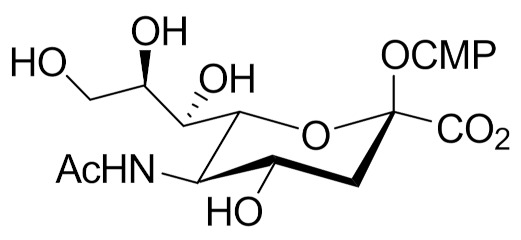 Donor (CMP-Neu5Ac)	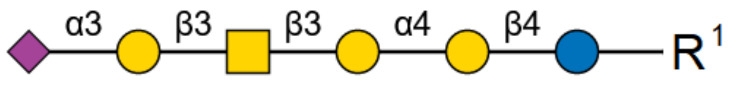 MSGb5	H	53	[124]
				PenNH_2_	69	
	
ST6GalNAc5	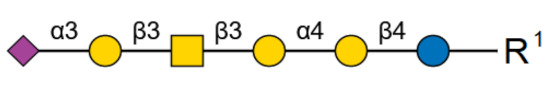 MSGb5		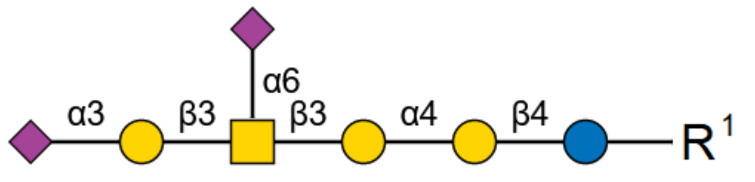 DSGb5	H	50	
				PenNH_2_	57	
	
ST6Gal1	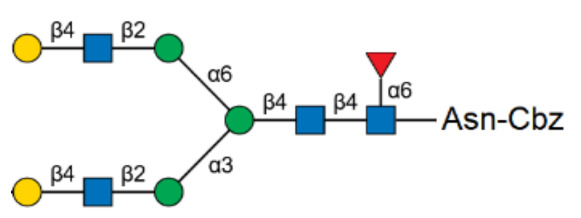 Bi-antennary N-glycan		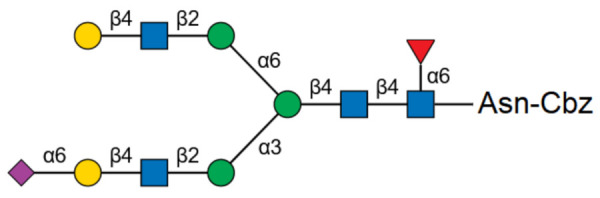 Mono-sialyl Bi-antennary N-glycan		68	[126]
ST3Gal1	Asialofetuin	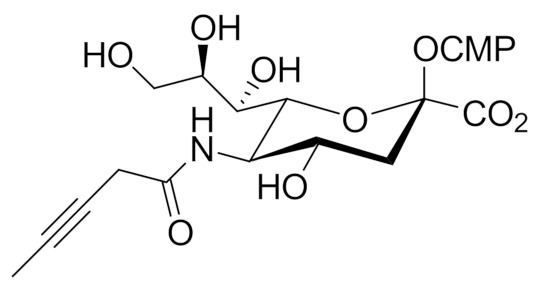 Donor	sialyl fetuin		No mention	[129]
ST6Gal1						
ST6Gal1		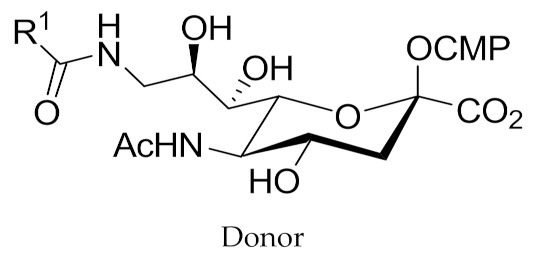 Donor		CH_2_N_3_	No mention	[131]
				PhSydCl		

ST3Gal4				CH_2_N_3_		

ST3Gal1	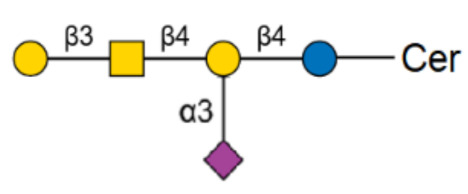 GM1a	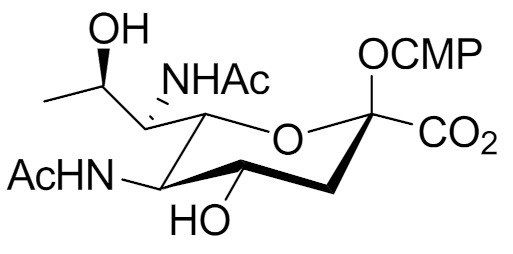 Donor (Leg5Ac7Ac)	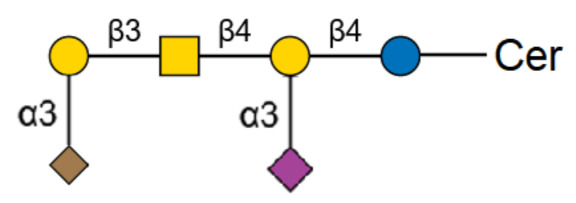 Leg-GD1a		No mention	[132]
	asialo-interferon-α2b		mono-Leg-interferon-α2b			
ST6Gal1	asialo-A1AT		tri-Leg-A1AT			
ST6Gal1	asialo-A1AT	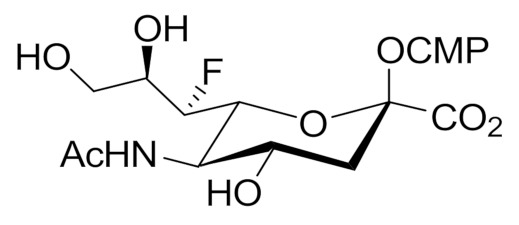 Donor	mono-sialyl-A1AT		No mention	[121]

**Table 4:** Examples of fluorescent labeling of cell-surface sialoglycans using recombinant SiaT.

SiaT	Fluorescence Tag	Labeled Osition in Sia	Cell Line Used for Labeling	Ref.
PmST1 (M144D)	Cy5	C5 acetamide	CHO cells	[108]
	Biotin			
Pd2,6ST	Cy5			
	Biotin			
NmPST	No tag		CHO cells chicken DF1 fibloblasts rat Schwann cells	[136]
ST3Gal1	Alexa Fluor 555	C9 hydroxyl	HeLa cells	[130]
ST6Gal1				
ST6GalNAc4				
ST3Gal1	BODIPY	C9 hydroxyl	Jurkat cells	[135]
ST6Gal1		C5 acetamide		
ST6Gal1	Biotin and heparan sulfate	C5 acetamide	Ext1^-/-^ cells	[139]
